# Identification of QTN-by-environment interactions for yield related traits in maize under multiple abiotic stresses

**DOI:** 10.3389/fpls.2023.1050313

**Published:** 2023-02-15

**Authors:** Yang-Jun Wen, Xinyi Wu, Shengmeng Wang, Le Han, Bolin Shen, Yuan Wang, Jin Zhang

**Affiliations:** ^1^ College of Science, Nanjing Agricultural University, Nanjing, China; ^2^ Key Laboratory of Crop Genetics and Germplasm Enhancement, Nanjing Agricultural University, Nanjing, China

**Keywords:** multiple abiotic stresses, QTN-by-environment interaction, GWAS, 3VmrMLM, yield-related traits, maize

## Abstract

**Introduction:**

Quantitative trait nucleotide (QTN)-by-environment interactions (QEIs) play an increasingly essential role in the genetic dissection of complex traits in crops as global climate change accelerates. The abiotic stresses, such as drought and heat, are the major constraints on maize yields. Multi-environment joint analysis can improve statistical power in QTN and QEI detection, and further help us to understand the genetic basis and provide implications for maize improvement.

**Methods:**

In this study, 3VmrMLM was applied to identify QTNs and QEIs for three yield-related traits (grain yield, anthesis date, and anthesis-silking interval) of 300 tropical and subtropical maize inbred lines with 332,641 SNPs under well-watered and drought and heat stresses.

**Results:**

Among the total 321 genes around 76 QTNs and 73 QEIs identified in this study, 34 known genes were reported in previous maize studies to be truly associated with these traits, such as ereb53 (GRMZM2G141638) and thx12 (GRMZM2G016649) associated with drought stress tolerance, and hsftf27 (GRMZM2G025685) and myb60 (GRMZM2G312419) associated with heat stress. In addition, among 127 homologs in Arabidopsis out of 287 unreported genes, 46 and 47 were found to be significantly and differentially expressed under drought vs well-watered treatments, and high vs. normal temperature treatments, respectively. Using functional enrichment analysis, 37 of these differentially expressed genes were involved in various biological processes. Tissue-specific expression and haplotype difference analysis further revealed 24 candidate genes with significantly phenotypic differences across gene haplotypes under different environments, of which the candidate genes GRMZM2G064159, GRMZM2G146192, and GRMZM2G114789 around QEIs may have gene-by-environment interactions for maize yield.

**Discussion:**

All these findings may provide new insights for breeding in maize for yield-related traits adapted to abiotic stresses.

## Introduction

Maize (*Zea mays*) is a vital and strategic cereal crop cultivated in a variety of agroecological zones across the world. Growing on non-irrigated fields exposes them to various environmental stresses, such as drought stress, heat stress, and their combination. Heat waves mixed with acute and persistent drought stress can have disastrous consequences for agriculture, as well as economic and social stability, especially affecting drylands utilized for grain production across the world (Ciais et al., 2005; Mittler, 2006; Zandalinas et al., 2020). The vulnerability of maize to drought and heat stresses can lead to yield losses of 15-20% every year (Khan et al., 2016). Such losses are likely to rise as a result of climate change, especially in emerging nations with rising maize consumption (Campos et al., 2006). To fulfill the future demands of the world’s rising population, high yielding and drought tolerant maize cultivars are seen as the most economically feasible answer (Monneveux et al., 2006).

Due to the poor heritability of grain production (Edmeades et al., 1999) and the likelihood of drought occurring at several growth periods, direct selection for grain yield under drought circumstances is frequently challenging (Chen et al., 2012). The use of secondary traits in breeding programs has become one of the finest methods for choosing the genotypes that perform the best under stress situations (Parajuli et al., 2018). Due to the separation of male and female flowers, maize is more vulnerable to drought than any other crop, especially when temperatures are rising above 35°C (Huang et al., 2006). Consequently, the rise in anthesis-silking interval is one of the primary effects of drought stress in maize (Bänziger et al., 2000). The anthesis date keeps a strong genetic correlation with grain yield and remains highly heritable and cost-effective to measure (Cerrudo et al., 2018). These studies demonstrated that the secondary traits comprising anthesis-silking interval and anthesis date have been included in breeding programs to promote indirect selection for grain yield.

As global climate change accelerates, quantitative trait nucleotide (QTN)-by-environment interactions (QEIs) play an increasingly essential role in the genetic dissection of complex traits in plants (Lukens and Doebley, 1999). There are currently accessible methodologies and software tools for identifying QEIs. Crossa et al. (1999) developed a factorial regression model for QEI in tropical maize. In its basic form, an additional covariate needs to be introduced for each putative QTL, thus least squares estimate approaches fail when there are a large number of genotypic or environmental covariables. To detect QEIs, Zhu and Weir (1998) and Wang et al. (1999) developed the mixed-model based composite interval mapping (MCIM) approach, but the results may be susceptible to the specified model of multiple QTL (Piepho, 2000). Li et al. (2015) expanded the inclusive composite interval mapping (ICIM) main-effect genetic model into a QEI model. In real data analysis, it is challenging to uncover small QEIs. However, these approaches are suitable in bi-parental segregation populations. Although Moore et al. (2019) proposed the structured linear mixed model (StructLMM) to detect QEIs, only allelic substitution was detected, and its polygenic background was controlled. To over these issues, recently, Li et al. (2022a, 2022b) proposed a compressed variance component mixed model (3VmrMLM) to detect and estimate all the effects in QTN and QEI detection under controlling all the possibly polygenic backgrounds in genome-wide association studies (GWAS). Based on a full mixed-model framework, the numbers of variance components in QTN and QEI detection were compressed from 5 and 10 to 3, respectively, showing very good performances in computational efficiency. Furthermore, 3VmrMLM can identify QTNs and QEIs accurately and estimate their genetic effects unbiasedly (Zuo et al., 2022; Zhao et al., 2023).

From now, lots of genes response to abiotic stresses were identified in *Arabidopsis*, rice and maize. For example, in *Arabidopsis*, *DREB2A* is one of the transcription factors that activates the expression of heat-stress-responsive genes (Sakuma et al., 2006a). *DREB2A* has a conserved ERF/AP2 DNA-binding domain and recognizes a dehydration-responsive element (DRE). This DRE was reported to function as a heat-stress-responsive element (Sakuma et al., 2006b). Liu et al. (2013a) reported that *di19* functions as a transcriptional regulator and is involved in *Arabidopsis* responses to drought stress through up-regulation of pathogenesis-related *PR1*, *PR2*, and *PR5* gene expressions. In rice, *OsGRAS23* can bind to the promoters of several target genes and modulate the expressions of a series of stress-related genes. Overexpression of *OsGRAS23* conferred transgenic rice plants with improved drought resistance (Xu et al., 2015). The RING finger ubiquitin E3 ligase *OsHTAS* functions in leaf blade to enhance heat tolerance through modulation of hydrogen peroxide-induced stomatal closure. In maize, *ZmHsf11* decreases plant tolerance to heat stress by negatively regulating the expression of oxidative stress-related genes, thus increasing reactive oxygen species levels and decreasing proline content. It is a negative regulator involved in high temperature stress response (Qin et al., 2022). In addition, the overexpression of *ZmPIS* in maize plants under drought stress might lead to the increased synthesis of unsaturated phospholipid and galactolipid species, which are involved in the maintenance of membrane permeability and fluidity that might contribute to plant adaptation to drought stress (Liu et al., 2013b). However, seldom maize gene-by-environment interactions (GEIs) were identified, most of the maize genes were identified by transcriptome analysis and comparative genome analysis (Shi et al., 2017; Zhao et al., 2019). Mining QEIs and related GEIs would provide excellent genes for the genetic improvement of high tolerance to biological stress breeding in maize.

In this study, 3VmrMLM was used to detect QTNs and QEIs for three yield-related traits in an association-mapping panel of 300 tropical and subtropical inbred maize lines each with 955,690 single nucleotide polymorphisms (SNPs) from the DTMA (Drought Tolerant Maize for Africa, https://www.cimmyt.org/projects/drought-tolerant-maize-for-africa-dtma/) in four environments. The transcriptomic data of drought treatment *vs.* well-watered and high *vs.* normal temperature, respectively, were used to identify differentially expressed genes. Functional enrichment, tissue-specific expression, and haplotype and phenotypic difference analysis were used to further validate the candidate maize genes in drought and heat stresses. Multi-environment joint analysis will be helpful for identifying candidate genes related to yield under multiple abiotic stresses in maize.

## Materials and methods

### Phenotypic data and statistical analysis

The DTMA panel datasets were achieved from International Maize and Wheat Improvement Center (CIMMYT, http://hdl.handle.net/11529/10548156), including 300 inbred lines of tropical and subtropical maize gathered and tested against CML-539 (Wen et al., 2011). Three yield-related traits, grain yield (GY, ton/hectare), anthesis date (AD, day), and anthesis-silking interval (ASI, day), were investigated to detect QTNs and QEIs. The yield trial data were collected from Mexico, Kenya, Thailand, Zimbabwe, and India between 2008 and 2011 under environments of well-watered (WW), drought stress (DS), heat stress (HS), and combined drought and heat stress (DHS). The detailed description and calculated best linear unbiased prediction values for each yield-related trait under the various scenarios were provided by Cairns et al. (2013).

To better understand the patterns of variation of three yield-related traits under various environments, we calculated Pearson correlation coefficients and carried out significance tests for 12 trait-environment combinations using *cor.test* function based on R (Version 4.2.1). The violin plots were adopted to illustrate the variation of three traits under four environments by using the *ggbetweenstats* function in ggstatsplot package of R (Patil, 2021), and the *Kruskal-Wallis* one-way analysis of variance by ranks was conducted with the parameter "type" set to "nonparametric" to test whether the phenotypic mean of each trait differed significantly across four environments.

### Genotypic data

We obtained the original genotypic data from http://hdl.handle.net/11529/10548156, with a total of 955,690 SNPs. Then we performed quality control on the SNP dataset by filtering markers with minor allele frequency (MAF) < 0.01 and missing genotype rate > 25% by PLINK (Version 1.9). The imputation of the absent markers was carried out by Beagle (Version 5.4) with the default settings (Browning et al., 2018). Ultimately, we obtained 332,641 SNPs with known physical positions and high quality for further research. To visualize the genotype in this study, PopLDdecay (Version 3.31, https://github.com/BGI-shenzhen/PopLDdecay) was used to calculate linkage disequilibrium (LD) on SNP pairs within a 10-kb window. In addition, the distribution of 332,641 SNPs across 10 chromosomes was plotted by CMplot package in R.

### GWAS method

We performed GWAS for the detection of QEIs and QTNs using the IIIVmrMLM package (https://github.com/YuanmingZhang65/IIIVmrMLM; Li et al., 2022b) in R, with high computational efficiency. It mainly used the *IIIVmrMLM* function, where the parameter "method" was set to "Multi_env". The kinship matrix was also calculated *via* the package. In the 3VmrMLM method, the P-value thresholds for significant and suggested QTNs or QEIs were based on Bonferroni correction (P-value < 0.05/*m*, where *m* is the number of markers) and logarithm of odds (LOD) score ≥ 3.0, respectively. In the following analysis, as long as one of them was satisfied, we considered it as QTNs or QEIs significantly associated with the target traits. In addition, the package can automatically generate the attractive Manhattan diagrams.

### Differential expression and functional enrichment analyses

**Figure 1:**
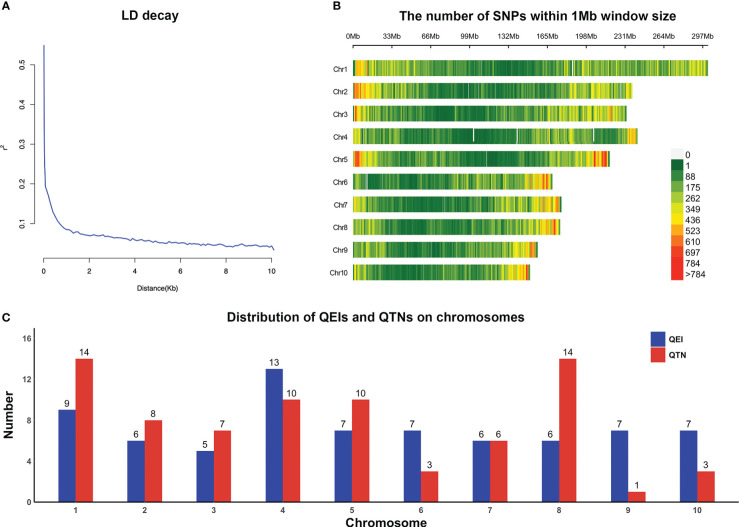
**(A)** LD decay plot for high-quality SNPs. **(B)** Distribution of high-quality SNPs on chromosomes. **(C)** Distribution of QEIs and QTNs across all chromosomes.

Genes situated within or contiguous 5 kb (5 kb upstream and downstream, total 10 kb, according to LD decay shown in **Figure 1A**) of the QTNs and QEIs significantly associated with the target traits were extracted following the B73 AGPV2 (MaizeGDB, https://www.maizegdb.org/) reference genome assembly (Woodhouse et al., 2021). The DNA sequence of all detected genes was used for similarity search on BLAST (https://blast.ncbi.nlm.nih.gov/Blast.cgi) in order to determine the *Arabidopsis* ortholog.

For the above *Arabidopsis* homologous genes, excluding the known genes reported in the literatures, we performed differential expression analysis of the series GSE124340 and GSE154373 from the Gene Expression Omnibus (GEO, https://www.ncbi.nlm.nih.gov/geo/) database for the unreported genes to identify differentially expressed genes (DEGs) responding to drought stress and heat stress, respectively. The series GSE124340 contains transcript per million (TPM) value of maize under well-watered condition (WW) and drought treatments (DT) at various levels (DT2, DT3, and DT4 represent soil moistures for maize plants were 30-35%, 20-25%, and 10-15% respectively). Each treatment has 2 biological replicates. Meanwhile, the series GSE154373 contains fragments per kilobase of feature per million (FPKM) values for maize plants (inbred line W22) at different temperature treatments (31°C, 33°C, 35°C, and 37°C), with three replicates for each treatment. DEGs between two pairwise samples (DT2 *vs*. WW, DT3 *vs*. WW, DT4 *vs*. WW, 33°C *vs.* 31°C, 35°C *vs.* 31°C, and 37°C *vs*. 31°C) were discovered by limma package in R, with a cutoff of the absolute value of log_2_FoldChange greater than 1 and P-value less than 0.05. Simultaneously, these DEGs responding to drought stress and heat stress were intersected with the detected genes, respectively, and thus we obtained the DEGs responding to multiple abiotic stresses for yield-related traits.

For gene ontology-based functional enrichment analysis, information of the above DEGs related to traits were simultaneously submitted to the web-based program AgriGO (Tian et al., 2017). We performed singular enrichment analysis and *Fisher's exact* test with P-value less than 0.05 to select enrichment gene ontology (GO) terms (Xu et al., 2014).

### Tissue-specific expression, analysis of haplotype and phenotypic difference, and identification of candidate genes

The database MaizeGDB (https://www.maizegdb.org/) was used to investigate the expression of genes in various tissues to illustrate the association between genes enriched in significant pathways and phenotypic variations. The HaploView software (Version 4.1) was used to perform linkage disequilibrium and haplotype block studies, as well as estimate the frequency of haplotype populations in genes widely expressed in various tissues of maize (Barrett et al., 2005), for validating the associated loci between genes and traits. Significant variants were utilized for haplotype division for each gene, and phenotypic differences across haplotypes were examined using the *t.test* function in R. Genes with significant differences in phenotypes across haplotypes under different environments were considered as the candidate genes.

## Results

### Phenotypic variation and correlation

**Figure 2:**
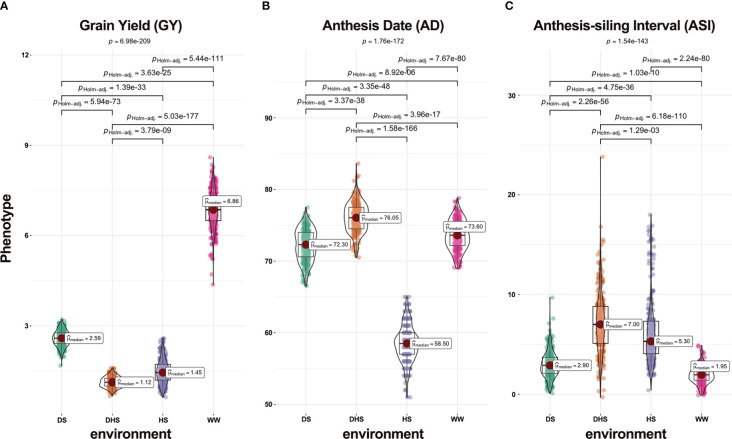
Violin plots of phenotypic distribution of three yield-related traits **(A)** grain yield (GY, ton/hectare), **(B)** anthesis date (AD, day), and **(C)** anthesis-silking interval (ASI, day) under the four evaluation conditions, i.e., drought stress (DS), combined drought and heat stress (DHS), heat stress (HS), and well-watered (WW).

The phenotypic performance of each trait varied under each environment, suggesting that the DTMA panel seemed to have large variation (**Figure 2**). All three traits examined under WW condition performed much better than those under stress situations including DS, HS, and DHS. The average performance for trait GY was much higher under WW than under all other situations (**Figure 2A**). On the other hand, the phenotypic variations for traits AD and ASI measured under WW were smaller than those under stress situations (**Figures 2B, C**). Except for DHS condition, the average value of AD was larger under WW than that under stress conditions (**Figure 2B**). The mean ASI value under WW was, however, smaller than that under stress conditions (**Figure 2C**). The P-values in the *Kruskal-Wallis* test for all three traits under four different environments were 6.98E-209, 1.76E-172, and 1.54E-143, respectively, and the P-values in any *pairwise comparison* test were less than 1.29E-03 (**Figure 2**), indicating that mean phenotypic values significantly differ across environments.

The phenotypic correlations among all yield-related traits under the same environment varied (**Supplementary Figure 1**). The correlations for GY under diverse situations were slight, favorable, and significant especially under WW. The correlations were favorable and extremely significant for AD between all situations. Only WW, DS, and HS had significant phenotypic correlations with ASI, while ASI under DHS was strongly linked with DS. On the whole, GY was negatively and strongly correlated with ASI under each condition, with a range of -0.67 to 0.08, confirming the previous findings (Ribaut et al., 2009). Nevertheless, none significant associations were found between GY and AD, or between AD and ASI under the same condition.

The phenotypic correlations between the same traits under various environments also varied (**Supplementary Figure 1**). For AD, the correlations between any two situations fluctuated from 0.55 to 0.95. The majority of correlations for GY and ASI under diverse situations varied from 0.09 to 0.60. The trait GY under DHS was not strongly correlated with DS or HS circumstance; furthermore, indirect correlations were observed between GY under DHS and that under DS or HS. The trait ASI under WW was positively correlated with DS or HS situation, but ASI under HS was uncorrelated with DHS situation.

Combined with the above analysis shown in **Figure 2** and **Supplementary Figure 1**, it can be justified that the DTMA panel is suitable for application in multi-environment joint analysis.

### Multi-environment joint analysis using 3VmrMLM

In total, 300 inbred lines with 332,641 SNPs were applied to carry out GWAS for each of three traits jointly analyzed in the four environments. LD decay measured the physical distance at which the Pearson’s correlation efficient dropped to half of the maximum (**Figure 1A**). These SNPs were evenly distributed across the 10 chromosomes (**Figure 1B**). The 3VmrMLM method used in this study identified 73 QEIs (57 significant and 16 suggested QEIs, **Supplementary Table 1**) and 76 QTNs (64 significant and 12 suggested QTNs, **Supplementary Table 2**) that were strongly associated with the yield-related traits.

In general, these QEIs and QTNs were distributed on all chromosomes (**Figure 1C**). For QEIs, the loci were spread out relatively evenly on the chromosomes, it was most distributed on chromosome 4 with 13 and least distributed on chromosome 3 with only 5 (**Figure 1C**). The highest number of QTNs was found on chromosomes 1 and 8, and the least on chromosome 9 (**Figure 1C**). On chromosomes 4 and 8, there were relatively more QTNs as well as QEIs, suggesting that these two chromosomes have a greater effect on the genetic variation of yield-related traits; while on chromosome 6, there were twice as many QEIs as QTNs, which may implicate that chromosome 6 may be more susceptible to environmental influences (**Figure 1C**).

**Figure 3:**
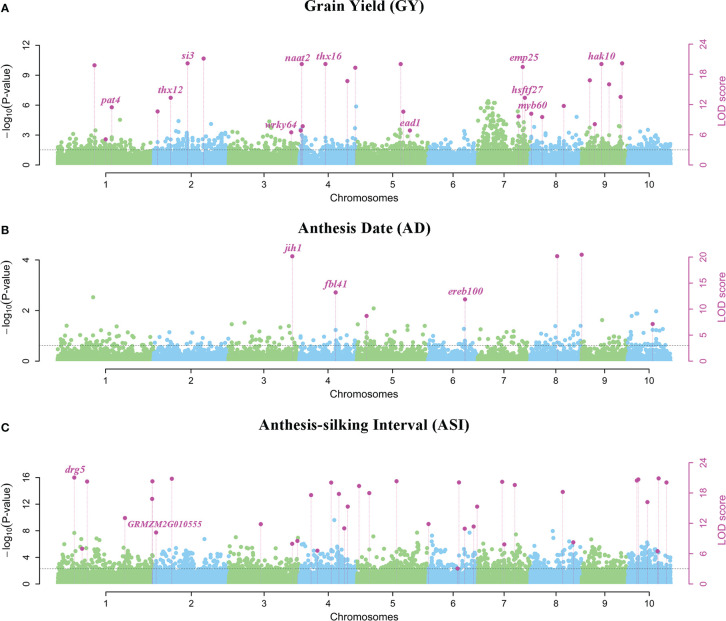
Manhattan plots using 3VmrMLM for QEIs on three yield-related traits **(A)** GY, **(B)** AD, and **(C)** ASI under four environments. Y-axis on the left side represents -log10 (P-values) of QEIs, which are obtained from single-marker genome-wide scanning for all markers, while y-axis on the right-side represents LOD scores, which are obtained from likelihood ratio test for QEIs, with the threshold of LOD = 3.0 (dashed line). These LOD scores are shown in points with straight lines. Highlighted text is the corresponding known gene of the loci.

A total of 29 QEIs were detected significantly related to GY, with P-values of 7.176E-129~8.065E-08 and LOD scores of 5.069~132.822, respectively (**Figure 3A**; **Supplementary Table 1**). Only 7 QEIs were distinguished for AD, with P-values of 6.123E-62~5.420E-10 and LOD scores of 7.130~65.274 (**Figure 3B**; **Supplementary Table 1**). The most QEIs were identified to be significantly associated with ASI in the multi-environment analysis, 37 QEIs were detected with P-values of 5.496E-121~1.978E-08 and LOD scores of 3.063~124.884 (**Figure 3C**, **Table 1**, and **Supplementary Table 1**).

**Table 1:** Results of 37 QEIs for trait ASI using multi-environment joint analysis of 3VmrMLM.

Marker	Chr	Pos (bp)	LOD (QEI)	add1	dom1	add2	dom2	add3	dom3	add4	dom4	r^2^(%)	P-value	SIG/SUG
S1_29787938	1	29787938	124.884	0.001	-1.342	-1.345	-4.045	1.172	8.005	0.172	-2.618	9.549	5.496E-121	SIG
S1_47457445	1	47457445	6.976	-0.145	0.062	-0.038	-0.140	0.355	0.112	-0.171	-0.034	0.412	1.544E-05	SUG
S1_62226889	1	62226889	46.714	-0.349	-2.227	0.110	-0.494	0.737	5.252	-0.498	-2.531	2.999	1.143E-43	SIG
S1_229206706	1	229206706	13.043	0.020	0.358	0.379	-0.340	-0.403	-0.332	0.004	0.313	0.776	4.369E-11	SIG
S1_297750016	1	297750016	16.830	-0.149	-0.672	-0.040	-0.636	0.363	2.123	-0.175	-0.816	1.029	1.172E-14	SIG
S1_298273269	1	298273269	51.094	0.297	-0.731	0.220	2.949	-0.919	-1.212	0.402	-1.006	3.317	5.696E-48	SIG
S2_2682470	2	2682470	10.180	0.109	-0.002	-0.396	-0.809	0.102	0.371	0.185	0.439	0.599	1.978E-08	SIG
S2_23529006	2	23529006	101.660	0.647	-1.729	0.382	-0.275	-1.446	4.059	0.417	-2.055	7.393	6.103E-98	SIG
S3_147588583	3	147588583	11.834	-0.151	0.284	0.344	0.666	-0.116	-1.342	-0.076	0.392	0.698	5.856E-10	SIG
S3_218123483	3	218123483	7.944	0.010	-0.290	-0.085	-1.586	0.255	2.819	-0.180	-0.943	0.468	2.126E-06	SUG
S3_226979707	3	226979707	8.521	0.067	-0.221	0.301	0.417	-0.321	-0.015	-0.047	-0.181	0.502	6.430E-07	SUG
S4_35625212	4	35625212	17.580	0.197	-1.786	0.083	-0.119	-0.347	4.782	0.067	-2.877	1.055	2.269E-15	SIG
S4_73208150	4	73208150	6.586	-0.056	-0.171	0.301	0.701	-0.131	0.392	-0.115	-0.923	0.385	3.405E-05	SUG
S4_167022069	4	167022069	25.660	-0.044	-0.660	-0.630	-0.003	0.314	-0.027	0.360	0.690	1.566	3.958E-23	SIG
S4_186691903	4	186691903	17.815	-0.031	-0.259	-0.070	-2.715	0.226	2.474	-0.125	0.500	1.069	1.355E-15	SIG
S4_202589250	4	202589250	11.007	-0.211	0.188	-0.047	0.257	0.353	-1.050	-0.095	0.605	0.652	3.426E-09	SIG
S4_223836871	4	223836871	15.310	-0.083	0.063	0.513	0.471	-0.260	-0.125	-0.169	-0.408	0.912	3.224E-13	SIG
S5_2353940	5	2353940	19.387	-0.006	-0.800	0.094	0.025	-0.020	2.037	-0.068	-1.263	1.213	4.279E-17	SIG
S5_14841812	5	14841812	17.978	-0.062	1.120	0.415	0.264	-0.222	-2.051	-0.131	0.667	1.078	9.482E-16	SIG
S5_160123104	5	160123104	52.683	0.524	1.284	-0.732	-2.472	-0.296	-0.251	0.503	1.439	3.378	1.561E-49	SIG
S6_656139	6	656139	11.863	0.154	-0.321	-0.450	0.359	0.226	0.150	0.070	-0.188	0.700	5.511E-10	SIG
S6_137397546	6	137397546	3.063	-0.108	-0.657	0.071	0.423	0.107	-0.297	-0.070	0.531	0.178	2.850E-02	SUG
S6_141276881	6	141276881	29.009	-0.336	-2.635	0.409	0.341	0.041	4.907	-0.114	-2.612	1.776	2.257E-26	SIG
S6_152209037	6	152209037	10.937	0.174	-1.475	-0.212	4.005	-0.117	-1.473	0.155	-1.056	0.576	3.975E-09	SIG
S6_163662312	6	163662312	11.361	0.156	-1.277	-0.004	3.920	-0.096	-0.857	-0.056	-1.785	0.671	1.611E-09	SIG
S6_167325529	6	167325529	15.302	0.010	0.262	-0.459	-0.552	0.383	0.335	0.067	-0.045	0.914	3.280E-13	SIG
S7_126213664	7	126213664	40.770	0.345	-0.367	-0.475	-1.435	-0.499	1.477	0.629	0.326	2.579	7.667E-38	SIG
S7_130495196	7	130495196	7.833	0.015	0.372	-0.281	-0.274	0.232	-0.692	0.033	0.594	0.461	2.672E-06	SUG
S7_155070876	7	155070876	19.580	-0.153	-0.057	0.572	0.977	-0.228	-0.707	-0.190	-0.213	1.176	2.802E-17	SIG
S8_147292704	8	147292704	18.210	0.064	-2.425	-0.194	5.252	0.304	-0.051	-0.173	-2.776	0.975	5.690E-16	SIG
S8_165163196	8	165163196	8.237	-0.086	0.041	-0.173	-0.394	0.373	0.396	-0.114	-0.044	0.486	1.160E-06	SUG
S10_34023703	10	34023703	66.109	-0.039	-0.783	1.082	-0.277	-0.433	2.405	-0.610	-1.345	4.410	9.192E-63	SIG
S10_50775539	10	50775539	87.805	0.109	-0.783	-1.280	-0.277	0.357	2.405	0.814	-1.345	6.153	3.260E-84	SIG
S10_100028483	10	100028483	16.198	-0.224	-2.458	0.196	0.686	0.212	4.198	-0.184	-2.426	0.967	4.663E-14	SIG
S10_133408126	10	133408126	6.483	-0.243	0.140	0.079	0.168	0.265	-0.509	-0.101	0.201	0.379	4.187E-05	SUG
S10_135046780	10	135046780	108.758	0.260	-2.433	0.665	0.778	-1.480	3.735	0.555	-2.080	8.053	5.569E-105	SIG
S10_145183843	10	145183843	27.872	-0.174	0.346	0.583	-0.734	0.137	0.020	-0.546	0.367	1.711	2.858E-25	SIG

Chr, chromosome; Pos, position; LOD, logarithm of odds; addk, additive effect in environment k; domk, dominance effect in environment k; r2 (%): the proportion of total phenotypic variance explained by each QEI. SIG, significant; and SUG, suggested.

On the other hand, numbers of the significantly associated QTNs of each trait under four environments varied from 20 for ASI to 34 for AD (**Supplementary Figure 2**, **Supplementary Table 2**). 22 QTNs related to GY were detected with P-values of 6.021E-30~9.862E-08 and LOD scores of 5.886~29.221(**Supplementary Figure 2A**, **Supplementary Table 2**). 34 QTNs were associated with AD, with P-values of 1.414E-41~8.291E-08 and LOD scores of 3.387~40.851(**Supplementary Figure 2B**, **Supplementary Table 2**), and moreover, 20 QTNs associated with ASI were detected with P-values of 3.386E-32~2.295E-08 (**Supplementary Figure 2C**, **Supplementary Table 2**). The loci S1_18891169 and S5_205942859 were also identified for AD in the previous study (Yuan et al., 2019).

Meanwhile, the total phenotypic variance explained (PVE) of QEIs for ASI was 71.214% (**Table 1** and **Supplementary Table 1**), higher than the PVE of QTNs 8.966% (**Supplementary Table 2**). Among these 37 QEIs, S1_29787938 located on chromosome 1 had the maximum PVE of 9.549% (**Table 1** and **Supplementary Table 1**). Although the PVE of QTNs for GY was relatively low at 0.515%, the PVE of QEIs was nearly four times higher at 1.974% (**Supplementary Tables 1**, **2**). For AD, the PVE of QTNs was 2.659%, which was higher than the PVE of QEIs (**Supplementary Tables 1**, **2**).

The dominance and additive effects for ASI were relatively significant in all four environments, as listed in **Table 1** and **Supplementary Table 1**. The interaction effect of dominance with the third environment HS for ASI was generally large, with an effect of 8.005 for S1_29787938 located on chromosome 1 and an effect of 4.907 for S6_141276881 located on chromosome 6 (**Table 1** and **Supplementary Table 1**). The interaction effect of additive effect with the first environment DS for AD was positive and moderate, S9_567464 located on chromosome 9, where its effect was 0.488 (**Supplementary Table 1**). For ASI, the interaction effect of additive with environment DS was also relatively high, the effect of S2_23529006 was 0.647, simultaneously, the effect of S5_160123104 was 0.524 (**Table 1** and **Supplementary Table 1**). In summary, the higher effect of interaction with the environment indicated that the effect of heat and drought stresses on crop yield is not negligible.

### Known genes around QEIs and QTNs for yield-related traits under multiple abiotic stresses

In multi-environment joint analysis, a total of 321 genes (5 kb upstream and downstream) were found to be around their significant loci based on MazieGDB against the B73 AGPV2 genome. 161 out of 321 genes were homologous to *Arabidopsis* and their functional annotations were listed in **Supplementary Table 3**. Number of genes varied among the three traits. In total, 117, 78, and 126 genes were found to be around the significant loci for GY, AD, and ASI, respectively (**Supplementary Table 3**). For ASI, 74 and 52 genes were found to be around QEIs and QTNs, respectively. At the same time, 63 and 54 genes were found to be around QEIs and QTNs for GY, respectively. However, for AD, 58 genes were found to be around QTNs, but only 20 were found to be around QEIs (**Supplementary Table 3**). Highlighting in **Figure 3** and **Supplementary Figure 2**, 34 known genes were annotated according to the previous literatures (Augustine et al., 2016; Qi et al., 2017; Li et al., 2019).

For QEIs, 11 known genes related to GY, 3 known genes related to AD, and 2 known genes related to ASI were identified (**Figure 3**; **Supplementary Table 3**). The known genes *thx12* (*GRMZM2G016649*, around the locus S2_21790763) and *thx16* (*GRMZM2G063203*, around the locus S4_149899538) related to GY (**Figure 3A**; **Supplementary Table 3**) are Trihelix transcription factors (also known as GT transcription factors) that are unique to plants and play important roles in abiotic drought stress (Du et al., 2016). The known gene *hsftf27* (*GRMZM2G025685*) around the locus S7_169176208 (**Figure 3A**; **Supplementary Table 3**), which acts as a heat shock transcription factor, helps to resist many environmental stresses and is involved in the regulation of primary metabolism, was also related to GY (Haider et al., 2021). Moreover, the expression of known gene *myb60* (*GRMZM2G312419*) around the locus S8_2763002 (**Figure 3A**; **Supplementary Table 3**) in response to jasmonic acid was up-regulated in heat-tolerant maize variety, which is considered to be important signaling substances with respect to plant stress responses (Wang et al., 2020). The known gene *ead1* (*GRMZM2G329229*) around the locus S5_194560419 (**Figure 3A**; **Supplementary Table 3**) plays a critical role in malate-mediated female inflorescence development and provides a promising genetic resource for enhancing maize grain yield (Pei et al., 2022). Moreover, *emp25* (*GRMZM2G312954*, around the locus S7_166553957) (**Figure 3A**; **Supplementary Table 3**) functions in the splicing of *nad4* introns, and is essential to maize kernel development (Xiu et al., 2020). The known gene *ereb100* (*AC209257.4_FG006*) around the locus S6_153235783 related to AD (**Figure 3B**; **Supplementary Table 3**) belongs to the APETALA2/Ethylene-responsive factor (AP2/ERF), which plays an active role in growth, development, and adaptation to abiotic stresses in maize (Zhang et al., 2022). *Drg5* (*GRMZM2G135877*, around the locus S1_29787938) related to ASI (**Figure 3C**; **Supplementary Table 3**) is shown to be rhythmically expressed under dark and light-dark cycles (Dong et al., 2020).

For QTNs, 3 known genes were related to GY (**Supplementary Figure 2A** and **Supplementary Table 3**), of which *dek2* (*GRMZM2G110851*, around the locus S1_299093763) is a pentatricopeptide repeat protein that affects the splicing of mitochondrial *nad1* intron 1 and is required for mitochondrial function and kernel development (Qi et al., 2017). Meanwhile, 9 known genes were detected for AD (**Supplementary Figure 2B** and **Supplementary Table 3**), among which *ereb53* (*GRMZM2G141638*, around the locus S3_166796324) and *ereb60* (*GRMZM2G131266*, around the locus S1_211326173), among the largest transcription factors in plants, were shown to exhibit differential expression patterns at different developmental stages in maize confirmed by the previous study (Zhang et al., 2022), especially in response to three different abiotic stresses, suggesting their important roles in abiotic stress tolerance (Zhang et al., 2022). A total of 7 known genes were found to be related to ASI (**Supplementary Figure 2C** and **Supplementary Table 3**), of which *bzip22* (*GRMZM2G043600*, around the locus S7_140710756) is a transcription factor from the basic leucine zipper family, and they are involved in stress responses and hormone signaling (Cao et al., 2019).

There were few overlapped genes detected for the different traits, indicating the genetic divergence between the traits. One common gene homologous to *Arabidopsis* observed for *GRMZM2G064159* between a QTN of GY and a QEI of AD (**Supplementary Table 3**). Only one known gene *naat2* (*GRMZM2G006480*) around the locus S4_3890824, which was confirmed to be related to GY, was overlapped between QTN and QEI (**Figure 3**; **Supplementary Figure 2**, and **Supplementary Table 3**). This finding showed the challenge of enhancing maize GY response to numerous abiotic stress tolerances at the same time. The more detailed information about the genes around QTNs and QEIs identified by the 3VmrMLM method can be referred to **Supplementary Table 3**.

### Response to multiple abiotic stresses and GO enrichment pathway

The differential expression analysis was used to determine the response of genes to DS and HS stresses. Among 127 homologs in *Arabidopsis* out of 287 unreported genes, 46 were identified as DEGs under DT *vs*. WW treatments and 47 were identified as DEGs under high temperature *vs*. normal temperature treatments. Among them, 29 DEGs were identified in both DS and HS tolerance (**Supplementary Table 4**). *GRMZM2G152549* was simultaneously found in six comparison groups (**Supplementary Table 4**), but it was lowly expressed under different levels of drought treatment relative to WW condition. The absolute value of log_2_FoldChange for *GRMZM2G016084* was as high as 205.14, followed by *GRMZM5G896082* and *GRMZM2G048836*, which had absolute values of log_2_FoldChange of 200.905 and 198.9, respectively (**Supplementary Table 4**). The two genes *GRMZM5G896082* and *GRMZM2G048836* were highly expressed after severe drought treatment and heat treatment (**Supplementary Table 4**).

**Figure 4:**
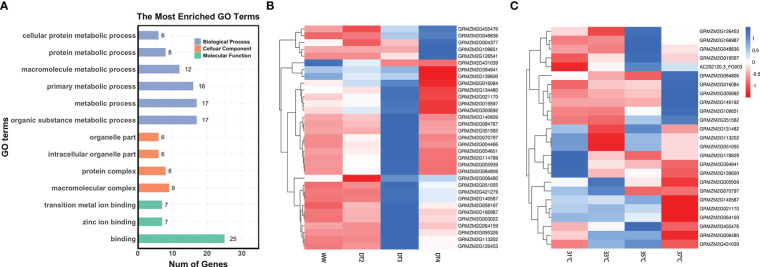
**(A)** Results of gene ontology-based functional enrichment analysis. **(B)** Clustered heatmap of expression values for 33 genes under different drought level treatments. WW stands for well-watered condition, DT2, DT3, and DT4 represent soil moistures for maize plants were 30-35%, 20-25%, and 10-15%, respectively. **(C)** Clustered heatmap of expression values for 25 genes under different temperature treatments (31°C, 33°C, 35°C, and 37°C). The numerical data represent the Z-score of mean TPM of two or three replicates.

According to outcomes of the GO functional enrichment analysis, a total of 37 genes among the above 46 and 47 DEGs significantly enriched to 13 GO terms associated with various biological processes (**Figure 4A**; **Supplementary Figure 3**, **4**). Such as, 17 genes around QEIs and QTNs were enriched to organic substance metabolic process (GO: 0071704), among which 2 genes *GRMZM2G109651* and *GRMZM2G048836* were also participated in the cellular component and molecular function (**Supplementary Figures 3** and **4**). Pleiotropic gene *GRMZM2G064159* which simultaneously identified around the locus S10_123819112, a QTN for GY and a QEI for AD was also involved in organic substance metabolic process (GO: 0071704, **Supplementary Figures 3** and **4**). Under adverse environment, plant metabolism is profoundly involved in signaling, physiological regulation, and defense responses (Fraire-Velázquez and Balderas-Hernández, 2013). Cellular components are the complex biomolecules and structures of which cells, and thus living organisms, are composed. In the last layer in **Supplementary Figure 3**, 6 genes were enriched to intracellular organelle part (GO: 0044446).

Moreover, the expression levels of some genes were significantly different under different treatment conditions. Under drought treatments (**Figure 4B**), most of the 33 genes were responded to drought stress. *GRMZM2G004377* around the locus S9_149252534, a QEI associated with GY, combined with candidate genes around the QEIs significantly associated with ASI such as *GRMZM2G140609*, *GRMZM2G084767*, and *GRMZM2G070797* had high expression under DT4 treatment and low expression under WW conditions (**Figure 4B**). In contrast, the gene *GRMZM2G431039* around the locus S7_155070876 associated with ASI had lower expression values under severe drought treatment and higher expression values under sufficient water conditions (**Figure 4B**). The expression levels of the 25 genes varied under different temperature treatments (**Figure 4C**). The gene *GRMZM2G146192* around the locus S4_2488289, a QEI associated with GY had a high expression value at 37°C, while *GRMZM2G178829* and *GRMZM2G139600* around QTNs significantly associated with AD had low expression values at high temperature (35°C and 37°C) (**Figure 4C**). A total of 21 genes responded to drought stress and heat stress, simultaneously (**Figures 4B, C**). Genes around QEIs significantly associated with ASI, such as *GRMZM2G016084* and *GRMZM2G084806*, were highly expressed under 37°C and DT3 treatment (**Figures 4B, C**). Gene *GRMZM2G02170* had low expression values under both high temperature at 37°C and extreme drought DT4 treatment (**Figure 4B, C**). In addition, some genes were expressed at different levels under drought stress and heat stress treatments. For example, the gene *GRMZM2G455476* had high expression value under DT4 treatment but low expression value under high temperature treatment at 37°C (**Figures 4B, C**). The gene *GRMZM2G070709* had high expression under DT3 treatment, but low expression value under high temperature treatment at 35°C (**Figures 4B, C**). This information may be useful in providing some biological basis for newly discovered heat and drought tolerant genes in maize.

### Haplotype and phenotypic difference analysis of candidate genes and tissue-specific expression profiles

Based on the results of tissue-specific expression, almost all the 37 genes significantly enriched to the pathways, except for *AC202120.3_FG003*, were expressed in various maize tissues. To further confirm the association between the genes and yield-related traits, we performed haplotype analysis of the remaining genes using SNPs within these genes and 2 kb upstream of them. A total of 24 genes differed significantly in phenotypes across haplotypes under different environments, and were considered as the candidate genes (**Table 2**). Among 24 candidate genes, there were 13 genes around QEIs and 13 genes around QTNs, with two candidate genes, *GRMZM2G006480* and *GRMZM2G064159*, being detected around both QEIs and QTNs. The more detailed results were listed in **Table 2** and **Supplementary Table 5**.

**Table 2:** Results of 24 candidate genes and functional annotation of *Arabidopsis* homologous genes.

Trait	QTN/QEI	Marker	Candidate Gene	Phytozome Annotations
GY	QEI	S4_2488289	*GRMZM2G146192*	beta-xylosidase 2
	QTN&QEI	S4_3890825	*GRMZM2G006480*	Tyrosine transaminase family protein
	QEI	S4_238951599	*GRMZM2G019597*	tRNA (guanine-N-7) methyltransferase
	QTN	S6_113109041	*GRMZM2G048836*	FTSH protease 6
	QEI	S7_160600156	*GRMZM2G058197*	C2H2-like zinc finger protein
	QEI	S9_47606538	*GRMZM2G131482*	surp domain-containing protein
	QEI	S9_149252534	*GRMZM2G004466*	seed storage 2S albumin superfamily protein
	QTN	S10_123819112	*GRMZM2G064159*	porphyromonas-type peptidyl-arginine deiminase family protein
AD	QTN	S1_279123888	*GRMZM2G351582*	ZPR1 zinc-finger domain protein
	QTN	S4_6553499	*GRMZM2G054651*	HVA22 homologue A
	QEI	S5_10542294	*GRMZM2G114789*	RNA-binding (RRM/RBD/RNP motifs) family protein
	QTN	S7_161438376	*GRMZM2G178829*	ARM repeat superfamily protein
	QTN	S7_174741307	*GRMZM2G134480*	ubiquitin activating enzyme 2
	QTN	S8_14796428	*GRMZM2G139600*	gamma-glutamyl transpeptidase 4
	QTN	S8_62998618	*GRMZM2G109651*	Cyclin/Brf1-like TBP-binding protein
	QEI	S10_123819112	*GRMZM2G064159*	porphyromonas-type peptidyl-arginine deiminase family protein
ASI	QEI	S1_47457445	*GRMZM2G300692*	galacturonosyltransferase-like 7
	QEI	S1_297750017	*GRMZM2G016084*	Nucleic acid-binding proteins superfamily
	QTN	S3_213937689	*GRMZM2G166987*	GDSL-like Lipase/Acylhydrolase superfamily protein
	QTN	S4_2764858	*GRMZM2G126453*	AAA-type ATPase family protein
	QEI	S6_141276882	*GRMZM2G084806*	Leucine-rich repeat protein kinase family protein
	QEI	S6_152209037	*GRMZM2G140587*	GDA1/CD39 nucleoside phosphatase family protein
	QEI	S6_167325529	*GRMZM2G051055*	casein kinase 1
	QTN	S10_96835918	*GRMZM2G021170*	Nucleic acid-binding OB-fold-like protein
	QTN	S10_127370470	*GRMZM2G005939*	basic helix-loop-helix DNA-binding superfamily protein

**Figure 5:**
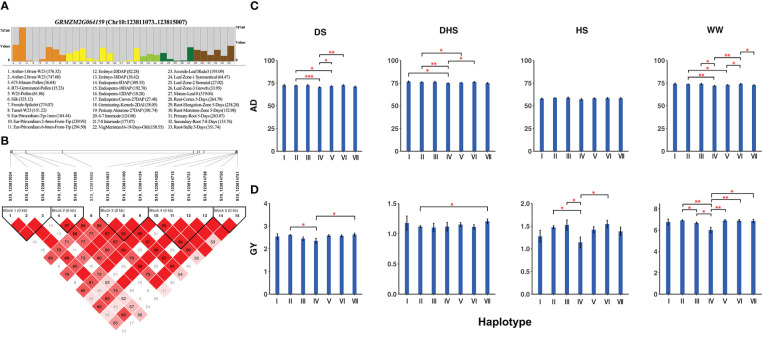
**(A)** Tissue-specific expression profile, **(B)** Linkage disequilibrium, and haplotype block with 14 SNPs inside for the candidate gene GRMZM2G064159. **(C)** Comparison of trait AD among haplotypes I (AACGCAACAGGACA), II (AACGCAGCGCCGCT), III (AACGCAGCGGCATA), IV (AAGGCAGCGCCGCT), V (AAGGCAGCGGCATA), VI (GCGGCAACAGGACA) and VII (GCGGCAGCGCCGCT). **(D)** Comparison of trait GY among haplotypes I, II, III, IV, V, VI, and VII. The number of stars represents the result of *t* test at different significance levels (*:0.05; **:0.01; ***:0.001).

Pleiotropic candidate gene *GRMZM2G064159* (CDS coordinates [5′-3′]: 123811073 ~ 123815007) around the locus S10_123819112, a QEI for AD and a QTN for GY (**Table 2**; **Supplementary Tables 3** and **5**), was analyzed to reveal the intragenic variation affecting the yield and to identify favorable haplotypes. **Figure 5A** exhibited the tissue-specific expression profile of the candidate gene *GRMZM2G064159*, which has a much higher expression value of 747.60 in Anther-2.0mm-W23 and is also commonly expressed in spike, embryo, and root-associated tissues. **Figure 5B** showed the linkage disequilibrium and haplotype block with 15 SNPs. The 300 inbred lines were classified into 7 haplotypes based on 14 SNPs (S10_123811034, S10_123811055, S10_123811069, S10_123811287, S10_123811289, S10_123814031, S10_123814100, S10_123814124, S10_123814202, S10_123814715, S10_123814731, S10_123814738, S10_123814750, S10_123814751). For AD, haplotype VI (GCGGCAACAGGACA) had the highest mean phenotypic values in DS (72.63) and DHS (76.17) conditions, whereas haplotype IV (AAGGCAGCGCCGCT) presented the lowest mean phenotypic values in DS (70.45) and DHS (74.48) conditions (**Figure 5C**). A *t* test showed that significant differences in DS condition existed between haplotypes II and IV (P-value = 4.62E-04, **Supplementary Table 5**). There was also a significant difference in DHS condition between haplotypes II and IV (P-value = 4.13E-03, **Supplementary Table 5**). For GY, haplotype VII (GCGGCAGCGCCGCT) had the highest mean phenotypic values in DS (2.63) and DHS (1.21) conditions, while haplotype IV had the lowest mean phenotypic values under DS (2.35) and HS (1.14) conditions (**Figure 5D**). A *t* test showed that significant differences in HS condition between haplotypes IV and VI (P-value = 1.21E-02, **Supplementary Table 5**). Therefore, we hypothesized that the candidate gene *GRMZM2G064159* may interact with environments for yield-related traits in maize.

The candidate gene *GRMZM2G146192* (CDS coordinates [5′-3′]: 2481257 ~ 2484641) was detected around the locus S4_2488289, a QEI for GY (**Table 2**; **Supplementary Tables 3** and **5**). **Supplementary Figure 5A** showed the tissue-specific expression profile of *GRMZM2G146192*, with higher expression values in root and leaf-associated tissues. **Supplementary Figure 5B, C** revealed the results of the haplotype block and phenotype difference. We inferred that the candidate gene *GRMZM2G146192* might also respond to various environment conditions for maize yield.

*GRMZM2G114789* (CDS coordinates [5′-3′]: 10541987 ~ 10545884) was also detected around the locus S5_10542293, a QEI for AD (**Table 2**; **Supplementary Tables 3** and **5**). **Supplementary Figure 6A** showed the tissue-specific expression profile of the candidate gene *GRMZM2G114789*, with higher expression values in root and embryo-associated tissues. **Supplementary Figures 6B, C** revealed the results of the haplotype block and phenotype difference. Haplotype II (CCGGCCCAAGGCT) had the highest mean phenotypic values in DS (75.27), DHS (77.12), HS (60.29), and WW (75.27) conditions, whereas haplotype V (TCGGCCCAAGGCT) presented the lowest mean phenotypic values in DS (69.56), DHS (74.88), HS (56.4), and WW (71.42) conditions. **Supplementary Figure 6C** showed significant differences in all conditions between haplotypes II and V, haplotypes II and VI (TCGGCCCAAGGTT), and haplotypes II and VII (TCGGCTTCAGGTT). Therefore, we inferred that the candidate gene *GRMZM2G114789* might be also a gene that interacted with environments related to yield in maize.

In summary, we supposed that the three candidate genes around QEIs mentioned above might have potential gene-by-environment interactions, including *GRMZM2G064159*, *GRMZM2G146192*, and *GRMZM2G114789*. In addition, some candidate genes around QTNs differed significantly in phenotypes across haplotypes under different environments (**Supplementary Table 5**). For example, the candidate gene *GRMZM2G166987* (CDS coordinates [5′-3′]: 213939500 ~ 213945050) identified around the QTN S3_213937689, which was significantly associated with ASI (**Table 2**; **Supplementary Table 3**), showed that its haplotype I (GAGGCAG) and haplotype III (GCTACAG) were significantly different to the phenotype under DS, HS, and DHS conditions by *t* test (**Supplementary Table 5**). However, whether these candidate genes around QTNs have gene-by-environment interactions for yield-related traits in maize needs to be further verified by new experiments.

## Discussion

### Tolerance to drought and heat stresses

Drought stress and heat stress are the most significant abiotic restrictions in the present and future climate change scenarios. Any additional rise in the frequency and severity of these stressors, either separately or in combination, would have a devastating impact on world agricultural yield and food security. Although they impede agricultural output at all phases of development, the level of damage during the blooming stage, particularly during the seed filling phase, is essential and causes significant yield losses. Cultivating climate-resilient crops is thus an efficient means of adapting to climate change.

We only obtained the transcriptomic data for drought stress and heat stress, and couldn’t obtain ones for combined drought and heat stress. Then, 46 and 47 DEGs were found to be significantly expressed under drought *vs.* well-watered treatments, and high *vs*. normal temperature treatments, respectively. Among them, 29 genes were identified in both DS and HS tolerance (**Supplementary Table 4**). However, most of the candidate genes did not show significant differences in combined drought and heat stress across haplotypes (**Supplementary Table 5**). This finding indicated that tolerance to individual stresses in maize is genetically distinct from tolerance to combined drought and heat stress, and tolerance to either stress alone does not confer tolerance to combined drought and heat stress, which was confirmed in the previous study (Cairns et al., 2013). Identification of genes tolerance to combined drought and heat stress will be the further work.

### Genetic basis for yield-related traits in maize

3VmrMLM identified 73 QEIs and 76 QTNs significantly associated with three yield-related traits under four environments in this study. The total PVE of all significant QEIs was 73.191%, which is six times that of QTNs (**Supplementary Tables 1** and **2**). Moreover, this study found a higher contribution by QEIs to total variation (PVE = 71.214%) than QTNs (PVE = 8.967%) for ASI (**Table 1**; **Supplementary Tables 1** and **2**). For ASI, 4 out of QEIs had a PVE value greater than 5% (**Table 1** and **Supplementary Table 1**). Among these four QEIs, *drg5* (*GRMZM2G135877*) around the locus S1_29787938 (r^2^ = 9.549%, **Table 1**; **Supplementary Tables 1** and **3**) is a known gene that has been verified by transcriptome analysis in the previous study (Dong et al., 2020).

The two known genes *thx12* (*GRMZM2G016649*) around the QEI S2_21790763 (P-value = 2.299E-11, LOD = 13.341, **Figure 3A**; **Supplementary Tables 1** and **3**) and *thx16* (*GRMZM2G063203*) around the QEI S4_149899538 (P-value = 8.289E-22, LOD =24.292, **Figure 3A**, **Supplementary Tables 1** and **43**), related to GY and homologous to the *Arabidopsis* gene *AT1G76890*, are the GT factors and play important roles in drought stress (Du et al., 2016). The mRNA expression levels of GT factors were determined for maize under drought stress. Moreover, the known gene *hsftf27* (*GRMZM2G025685*) around the QEI S7_169176208 (P-value = 1.996E-08, LOD = 13.335, **Figure 3A**; **Supplementary Tables 1** and **Supplementary Table 1** and **3**), which acts as a heat shock transcription factor, helps to resist many environmental stresses and is involved in the regulation of primary metabolism (Haider et al., 2021), was also related to GY. The expression of known gene *myb60* (*GRMZM2G312419*) around the QEI S8_2763002 (P-value = 2.331E-11, LOD = 10.176, **Figure 3A**; **Supplementary Tables 1** and **3**) in response to jasmonic acid is up-regulated in heat-tolerant maize variety, which is considered to be important signaling substances with respect to plant stress responses (Wang et al., 2020). *Thx12* and *thx16* exhibited high expression levels in immature leaves and at the base of two leaves stage. *Hsftf27* and *myb60* had higher expression values in root tissue at all stages. Roots and leaves are major tissues in coping with drought and heat stresses (Du et al., 2016).

In addition, the known gene *ereb60* (*GRMZM2G131266*) around the QTN S1_211326173 (P-value = 1.181E-08, LOD = 7.928, **Supplementary Figure 2B**, **Supplementary Tables 2** and **3**) significantly associated with AD exhibited obvious spatial and temporal expression profiles, specifically expressed in embryos (Zhang et al., 2022), implying that it was involved in maize growth and development regulation. The known gene *ereb53* (*GRMZM2G141638*) around the QTN S3_166796324 (P-value = 4.437E-11, LOD = 10.353, **Supplementary Figure 2B**, **Supplementary Tables 2** and **3**) significantly associated with AD was highly up-regulated after drought stress by transcriptome analysis (Zhang et al., 2022). The known gene *bzip22* (*GRMZM2G043600*) around the QTN S7_140710756 (P-value = 7.000E-13, LOD = 12.155, **Supplementary Figure 2C**, **Supplementary Tables 2** and **3**) significantly associated with ASI has been demonstrated to play essential roles in drought stress primarily through the ABA signal transduction pathway in the reported literature (Cao et al., 2019). This finding implied that the main effect of QTNs may also reflect an influence of environmental interactions.

Except for the above known genes, we also detected 24 new candidate genes in this study (**Table 2**). Among them, *GRMZM2G064159*, *GRMZM2G146192*, and *GRMZM2G114789* around QEIs have been shown the potential gene-by-environment interactions for yield-related traits in maize. First, *GRMZM2G064159* was a pleiotropic candidate gene which was simultaneously identified around the locus S10_123819112, a QEI for AD (P-value = 1.128E-05, LOD = 7.130, **Supplementary Table 1**) and a QTN for GY (P-value = 3.032E-18, LOD = 17.519, **Supplementary Table 2**). *GRMZM2G146192* was found to be around the locus S4_2488289, a QEI for GY (P-value = 2.058E-05, LOD = 6.835, **Supplementary Table 1**). *GRMZM2G114789* was found to be around the locus S5_10542293, a QEI for AD (P-value = 4.598E-07, LOD = 8.6818, **Supplementary Table 1**). Second, they are homologous to *Arabidopsis* (**Table 2**; **Supplementary Table 3**). *GRMZM2G146192* is homologous to *AT1G02640* (*BXL2*, **Table 2**; **Supplementary Table 3**), which increased enzymatic saccharification efficiency in *Arabidopsis* (Ohtani et al., 2018). *GRMZM2G064159* is homologous to *AT5G08170* (*EMB1873*, **Table 2**; **Supplementary Table 3**), which acted upstream of or within embryo development ending in seed dormancy. EMB genes encoded proteins with an essential function required throughout the life cycle (Muralla et al., 2011). *GRMZM2G114789* is homologous to the RNA-binding family protein *AT4G17720* (*BPL1*, **Table 2**; **Supplementary Table 3**) which contains classical RNA recognition motif domains and is implicated in the response to cytokinin (Marondedze et al., 2016). Third, they were DEGs under DT *vs*. WW treatments or under high *vs*. normal temperature treatments (**Figures 4B, C**; **Supplementary Table 4**), and *GRMZM2G064159* and *GRMZM2G146192* both involved in organic substance metabolic process (GO: 0071704, **Supplementary Figure 3**), *GRMZM2G114789* involved in binding (GO:0005438, **Supplementary Figure 3**). Moreover, their phenotypic differences across haplotypes were significant under four environments (**Figure 5C**; **Supplementary Figures 5C**, **6C**, and **Supplementary Table 5**). Lastly, *GRMZM2G064159* was commonly expressed in spike, embryo, and root-associated tissues (**Figure 5A**). High expression in embryo implies that it may be involved in maize growth and development regulation (Zhang et al., 2022). The root system is the primary site that perceives drought stress signals (Seo et al., 2009). Besides, *GRMZM2G146192* was highly expressed in root and leaf-associated tissues (**Supplementary Figure 5A**). *GRMZM2G114789* was expressed at various stages in root, leaf, internode, seed, and embryo-associated tissues, with higher expression values in root and embryo-related tissues (**Supplementary Figure 6A**). Therefore, we supposed that the candidate genes *GRMZM2G064159*, *GRMZM2G146192*, and *GRMZM2G114789* around QEIs may have gene-by-environment interactions for yield-related traits in maize, although new experiments such as functional validation are necessary to explore these novel GEI-trait associations. Although the results for known genes suggested that genes around QTNs may reflect an influence of environmental interactions (such as *ereb60*, *ereb53*, and *bzip22*, **Supplementary Figure 2B, C** and **Supplementary Table 3**), whether the candidate genes identified around QTNs in this study (**Table 2**) have gene-by-environment interactions needs to be further explored.

In addition, for ASI, the dominance effect in HS situation was positive and significant, ranging from -2.051% to 8.005%. In contrast, the dominance effect in DS situation was relatively negative and moderate, with a range mostly concentrated from -2.635% to 0.284% (**Table 1** and **Supplementary Table 1**). While on the other hand, the overall PVE of QTNs and QEIs significantly associated with GY were relatively low, largely clustered at 0.01% to 0.56% (**Supplementary Tables 1** and **2**). These findings suggested that trait GY and secondary trait ASI under abiotic stress would be regulated by small effect QTNs or QEIs that are dispersed across the genome in maize. This also suggested that it is relatively difficult to use marker-assisted selection to improve maize yield due to the complexity of traits under multiple environments. And in real data application, introducing secondary yield-related traits to assist maize breeding might be a good choice, which is also consistent with the findings in Bolaños and Edmeades (1996).

### Methods comparison

We also performed a single-environment analysis in the DTMA panel using the IIIVmrMLM package. The PVE of QTNs for ASI under each environment ranged from 50.25% to 58.04% (**Supplementary Table 6**), while the total PVE of QEIs for ASI in the multi-environment joint analysis was as high as 71.214% (**Table 1** and **Supplementary Table 1**). Moreover, 102 QTNs and 221 genes for ASI were detected in the single-environment approach, of which 5 QTNs overlapped with QEIs in the multi-environment joint analysis, and 11 genes overlapped (**Supplementary Tables 3** and **6**), of which one known gene *drg5* (*GRMZM2G135877*) was confirmed to be dark response gene in the previous literature (Dong et al., 2020). There were few overlapped loci detected in single- and multi-environment analyses, further illustrating that the yield-related traits in maize are complex and relatively susceptible to environmental influences. The more detailed results were listed in **Supplementary Table 6**. To address this issue, it is necessary to optimize the “SearchRadius” parameter.

Under the framework multiple-locus association studies, a few multi-year and multi-location GWAS methods are applicable for high-dimensional data analysis, and the DTMA panel with 332,641 SNPs has been seldom applied to reveal QEIs. Compared to the above single-environment analysis in 3VmrMLM, the significant loci overlapped fewer. We also compared 3VmrMLM with ICIM method (Li et al., 2015). Firstly, to reduce the computational burden, we used *Levene's* test (Brown and Forsythe, 1974) in R and set the threshold to 0.05 to downscale the DTMA dataset. That is because the ICIM method is very slow in handling high-dimensional dataset and *Levene's* test can be used to detect potential loci for heterogeneity of variances due to potentially interacting SNPs such as QTN-by-environment interactions. 58,000~71,000 significant markers for each trait were identified by *Levene's* test. Then, the linkage map was converted according to the ratio of genetic distance to physical distance of 1.296 cM/Mb (Guo et al., 2015). Finally, we performed a multi-environment joint analysis for the above data using the QTL IciMapping 4.2 software (Meng et al., 2015). A comparison was listed in **Supplementary Table 7**. The threshold was set to LOD (A) > 3 for additive QTLs and LOD (A by E) > 3 for additive QTLs by environment interactions in ICIM approach. 3vmrMLM detected more QTNs or QEIs than additive QTLs or additive QTLs by environment interactions. In particular, for ASI, 3VmrMLM detected 37 QEIs (PVE = 71.214%), but ICIM detected only 6 additive QTLs by environment interactions (PVE = 9.34%). 3VmrMLM added the polygenic effect and population structure to control the genetic background, thus it might be relatively close to the true genetic models of plants and animals. In addition, the computing time for GY, AD, and ASI ranged from 1~2 days, while 3VmrMLM consumed less than 7 hours for each trait, which took about one fourth of ICIM’s. 3VmrMLM reduces the dimensionality of SNPs by single-locus method, and constructs the multi-locus model based on the remaining markers, which decreases computational volume and computational complexity. In summary, 3VmrMLM presents well-performance results with higher statistical power, lower false positive rate and high computational efficiency, and it is a recommended method in multi-environment joint analysis.

## Conclusion

In this study, we identified QTN-by-environment interactions for three yield-related traits in maize under four abiotic stresses using the newly proposed 3VmrMLM method. A total of 73 QEIs and 76 QTNs were identified. Moreover, 34 known genes and 24 candidate genes were identified in the vicinity of QEIs and QTNs. Among 34 known genes, *ereb53* (*GRMZM2G141638*) & *thx12* (*GRMZM2G016649*), and *hsftf27* (*GRMZM2G025685*) & *myb60* (*GRMZM2G312419*) were confirmed to play important roles in drought and heat stresses, respectively, by transcriptome and bioinformatics analysis in previous maize studies. Among 24 candidate genes, 13 genes around QEIs and 13 genes around QTNs were validated functioning in drought and heat stresses by homologous genes miming, differential expression, functional enrichment, tissue-specific expression, and haplotype and phenotypic difference analysis in this study. Importantly, *GRMZM2G064159*, *GRMZM2G146192*, and *GRMZM2G114789* around QEIs may have gene-by-environment interactions for yield. These findings will facilitate the mining of genes involved in maize breeding under the abiotic stresses.

## Data availability statement

The original contributions presented in the study are included in the article/**Supplementary Material**. Further inquiries can be directed to the corresponding author.

## Author contributions

JZ conceived the study. JZ, Y-JW, and XW designed the experiment. XW, SW, and LH performed data analyses under the assistance or guidance from JZ and Y-JW. BS and YW contributed resources. Y-JW and XW wrote the manuscript with the participation of all authors. All authors contributed to the article and approved the submitted version.

## Supplementary material

Supplementary Figure 1 Pearson correlation coefficients and test for three yield-related traits under four environments in the DTMA panel. (Upper right) Pearson correlation coefficients, when the color is darker, the association is stronger; (Lower left) Pearson correlation test, the number of stars represents the different significance level (*: 0.05; **: 0.01; ***: 0.001). NS indicates non-significant.

Supplementary Figure 2 Manhattan plots using 3VmrMLM for QTNs on three yield-related traits **(A)** GY, **(B)** AD and **(C)** ASI under four environments. Y-axis on the left side represents -log10 (P-values) of QTNs, which are obtained from single-marker genome-wide scanning for all markers, while y-axis on the right-side represents LOD scores, which are obtained from likelihood ratio test for QTNs, with the threshold of LOD = 3.0 (dashed line). These LOD scores are shown in points with straight lines. Highlighted text is the corresponding known gene of the loci.

Supplementary Figure 3 Hierarchical tree graph of overrepresented GO terms in biological process category generated by singular enrichment analysis. Boxes in the graph represent GO terms labeled by their GO ID, term definition and statistical information. The significant (P-value < 0.05) and non-significant terms are marked with color and white boxes, respectively. The diagram, the degree of color saturation of a box is positively correlated to the enrichment level of the term. Solid, dashed, and dotted lines represent two, one, and zero enriched terms at both ends connected by the line, respectively.

Supplementary Figure 4 Expression map of GO for the 37 genes.

Supplementary Figure 5
**(A)** Tissue-specific expression profile, **(C)** Linkage disequilibrium, and haplotype block with 6 SNPs inside for the candidate gene GRMZM2G146192. **(C)** Comparison of trait GY among haplotypes I (GTCTCC), II (CTTGGC), III (CTCTCC), and IV (CACTCT). The number of stars represents the result of *t* test at different significance levels (*: 0.05; **: 0.01; ***: 0.001).

Supplementary Figure 6
**(A)** Tissue-specific expression profile, **(B)** Linkage disequilibrium, and haplotype block with 13 SNPs inside for the candidate gene GRMZM2G114789.. **(C)** Comparison of trait AD among haplotypes I (CCGGCCCAACACT), II (CCGGCCCAAGGCT), III (CCGGCCCAAGGTT), IV (TCGGCCCAACACT), V (TCGGCCCAAGGCT), VI (TCGGCCCAAGGTT), and VII (TCGGCTTCAGGTT). The number of stars represents the result of *t* test at different significance levels (*: 0.05; **: 0.01; ***: 0.001).
