# Supplementary figures and images for: Identification of QTN-by-environment interactions for yield related traits in maize under multiple abiotic stresses
Source: Front Plant Sci. 2023 Feb 15;14:1050313. doi: 10.3389/fpls.2023.1050313 (PMC9975332; doi:10.3389/fpls.2023.1050313)

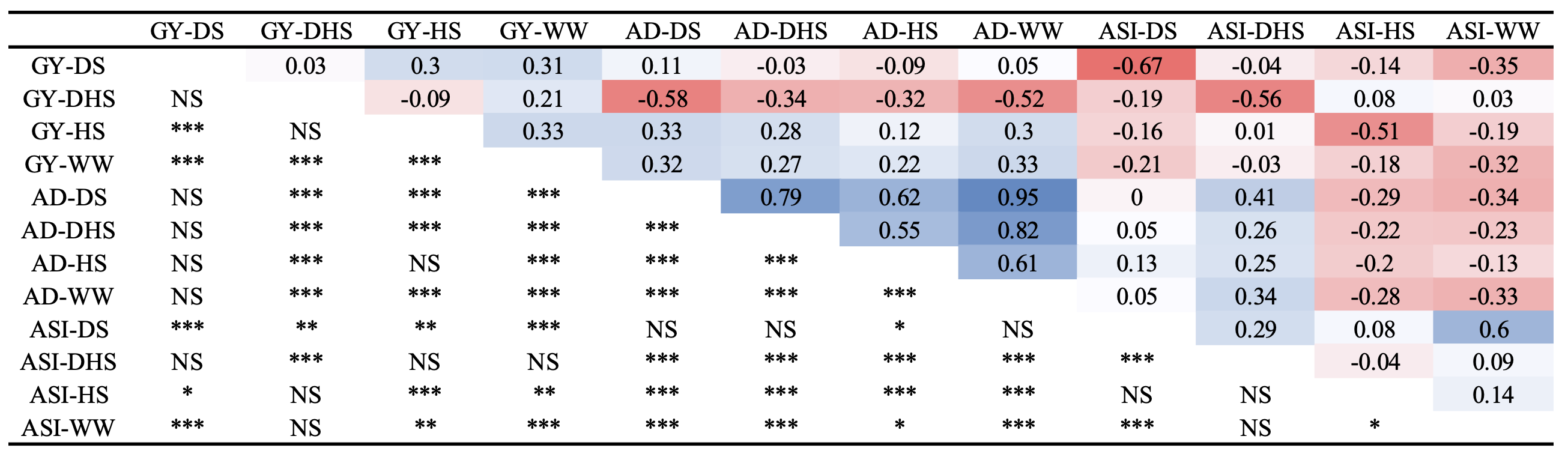

Supplement: Supplementary Figure 1 — Pearson correlation coefficients and test for three yield-related traits under four environments in the DTMA panel. (Upper right) Pearson correlation coefficients, when the color is darker, the association is stronger; (Lower left) Pearson correlation test, the number of stars represents the different significance level (*: 0.05; **: 0.01; ***: 0.001). NS indicates non-significant. [file Image_1.tif]

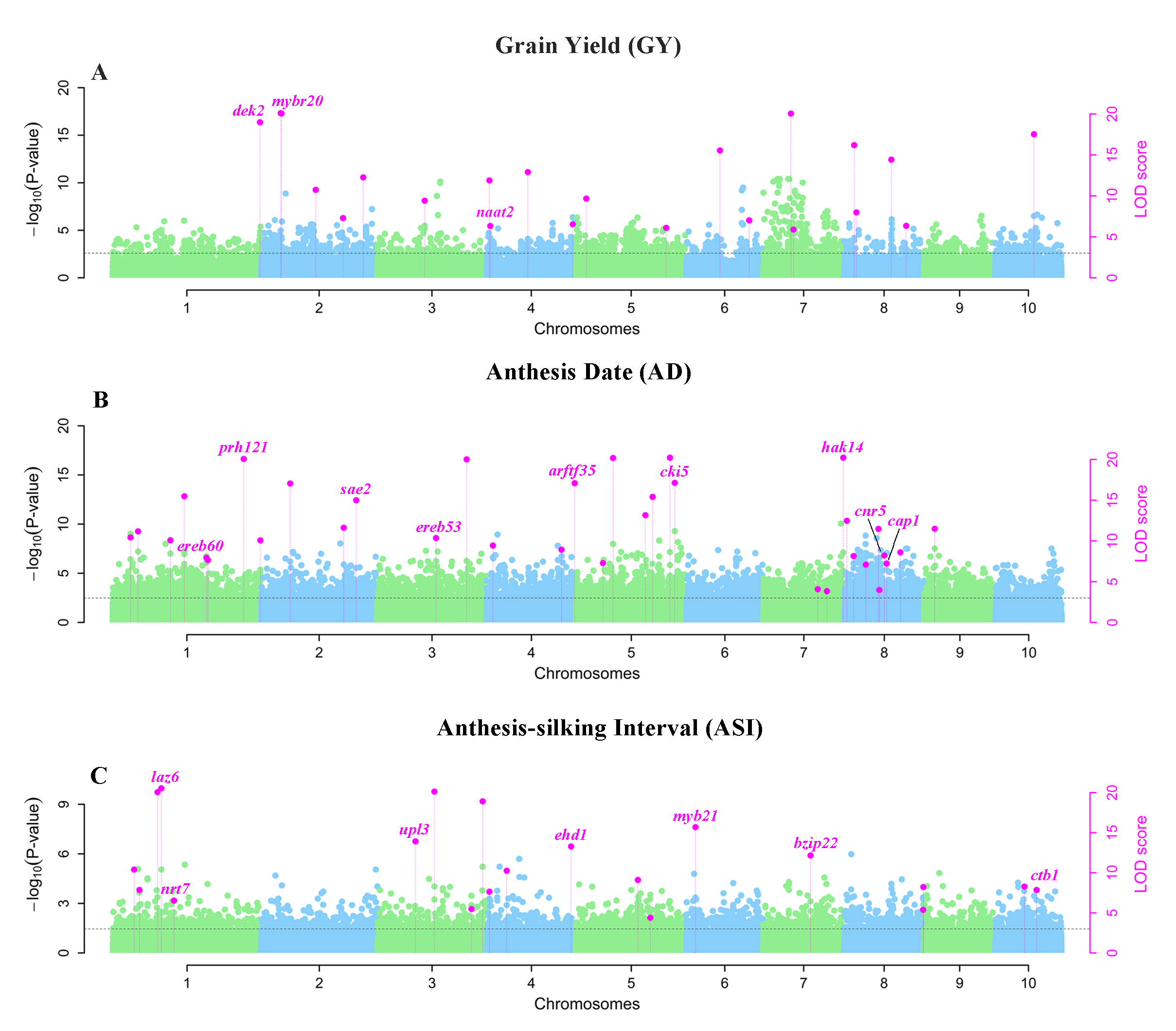

Supplement: Supplementary Figure 2 — Manhattan plots using 3VmrMLM for QTNs on three yield-related traits (A) GY, (B) AD and (C) ASI under four environments. Y-axis on the left side represents -log10 (P-values) of QTNs, which are obtained from single-marker genome-wide scanning for all markers, while y-axis on the right-side represents LOD scores, which are obtained from likelihood ratio test for QTNs, with the threshold of LOD = 3.0 (dashed line). These LOD scores are shown in points with straight lines. Highlighted text is the corresponding known gene of the loci. [file Image_2.tif]

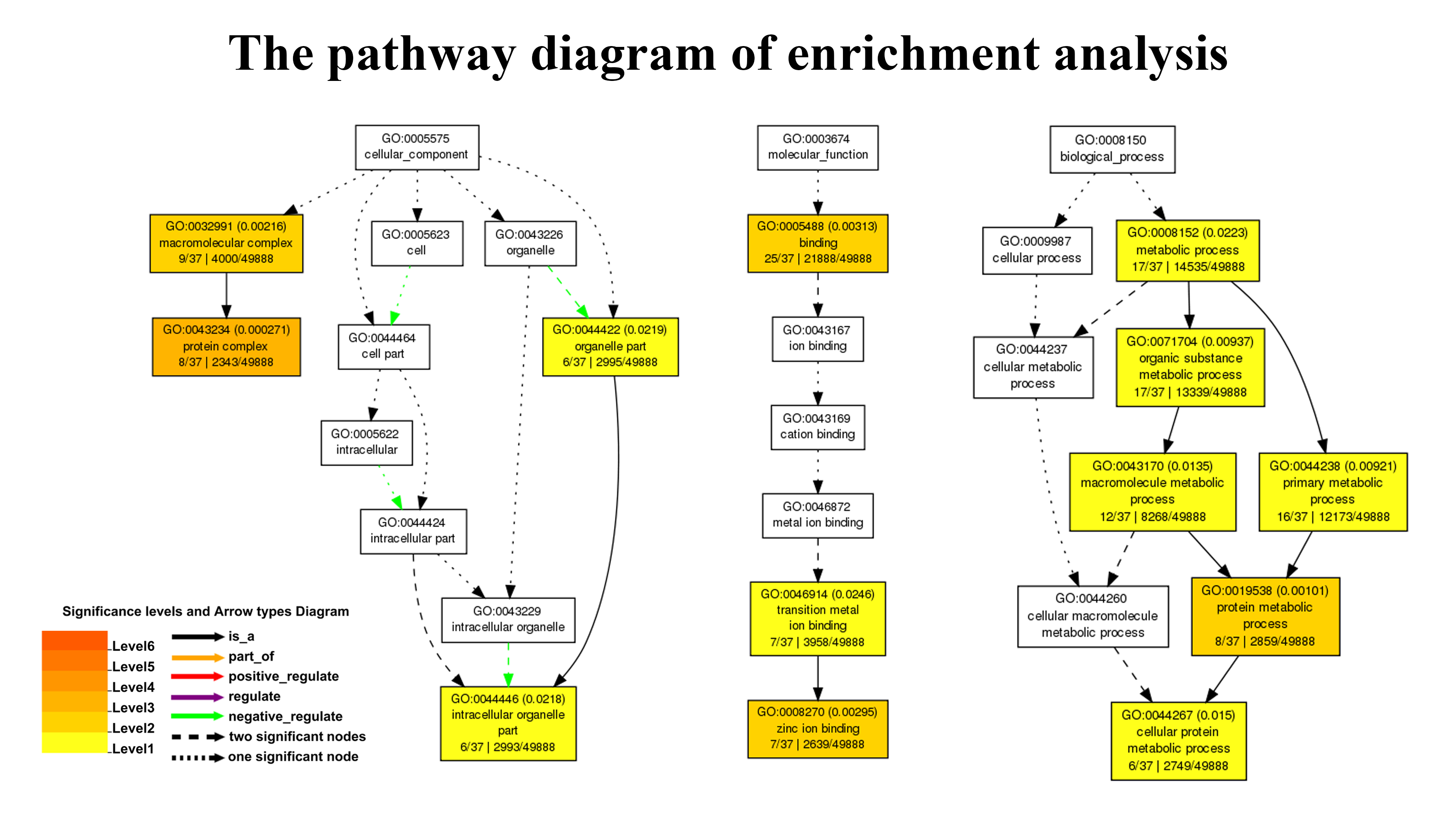

Supplement: Supplementary Figure 3 — Hierarchical tree graph of overrepresented GO terms in biological process category generated by singular enrichment analysis. Boxes in the graph represent GO terms labeled by their GO ID, term definition and statistical information. The significant (P-value < 0.05) and non-significant terms are marked with color and white boxes, respectively. The diagram, the degree of color saturation of a box is positively correlated to the enrichment level of the term. Solid, dashed, and dotted lines represent two, one, and zero enriched terms at both ends connected by the line, respectively. [file Image_3.tif]

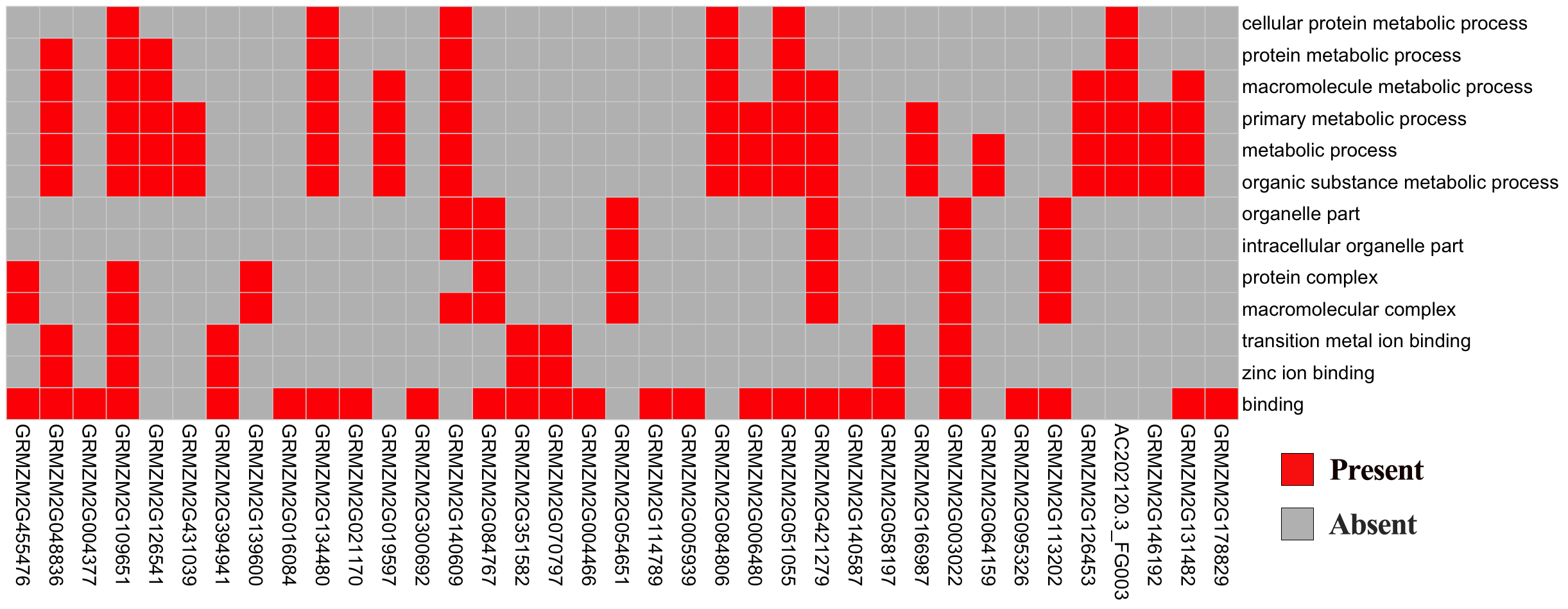

Supplement: Supplementary Figure 4 — Expression map of GO for the 37 genes. [file Image_4.tif]

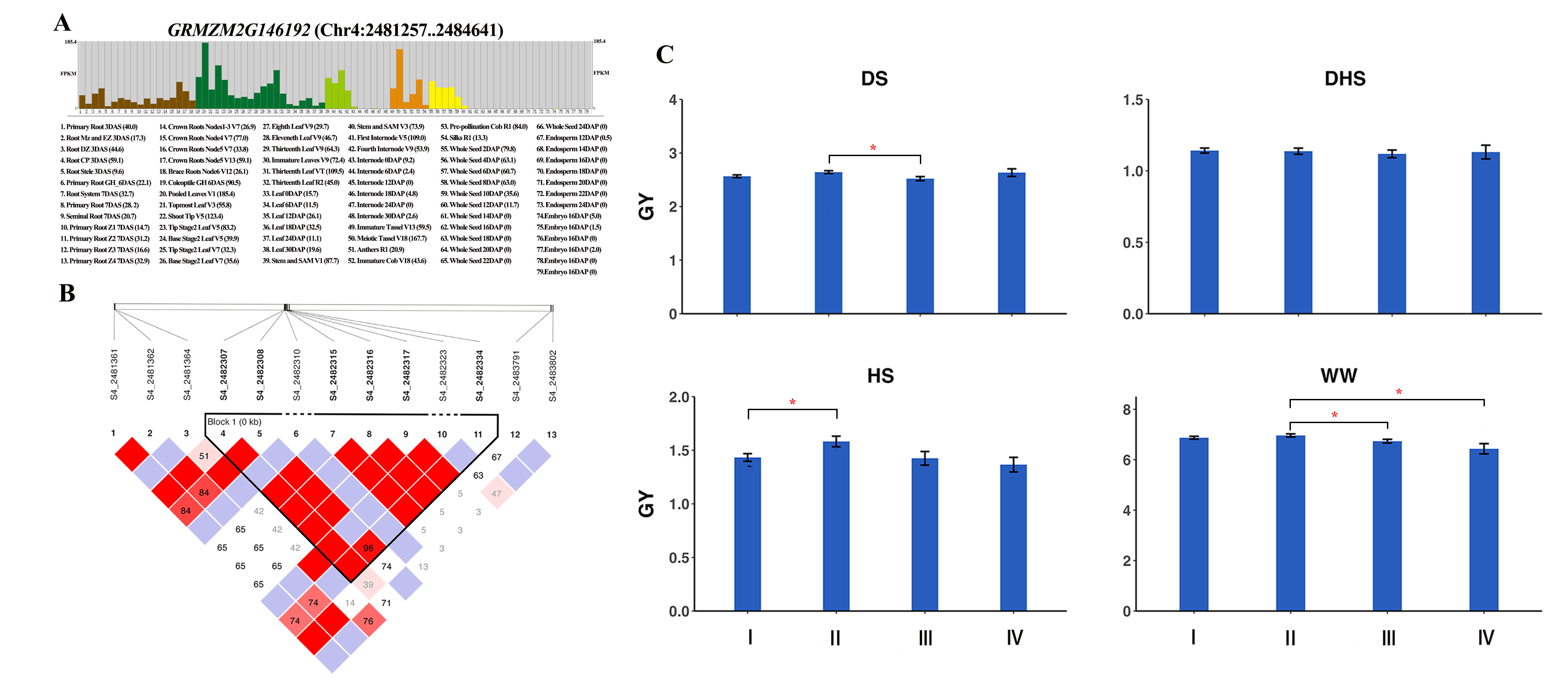

Supplement: Supplementary Figure 5 — (A) Tissue-specific expression profile, (C) Linkage disequilibrium, and haplotype block with 6 SNPs inside for the candidate gene GRMZM2G146192. (C) Comparison of trait GY among haplotypes I (GTCTCC), II (CTTGGC), III (CTCTCC), and IV (CACTCT). The number of stars represents the result of t test at different significance levels (*: 0.05; **: 0.01; ***: 0.001). [file Image_5.tif]

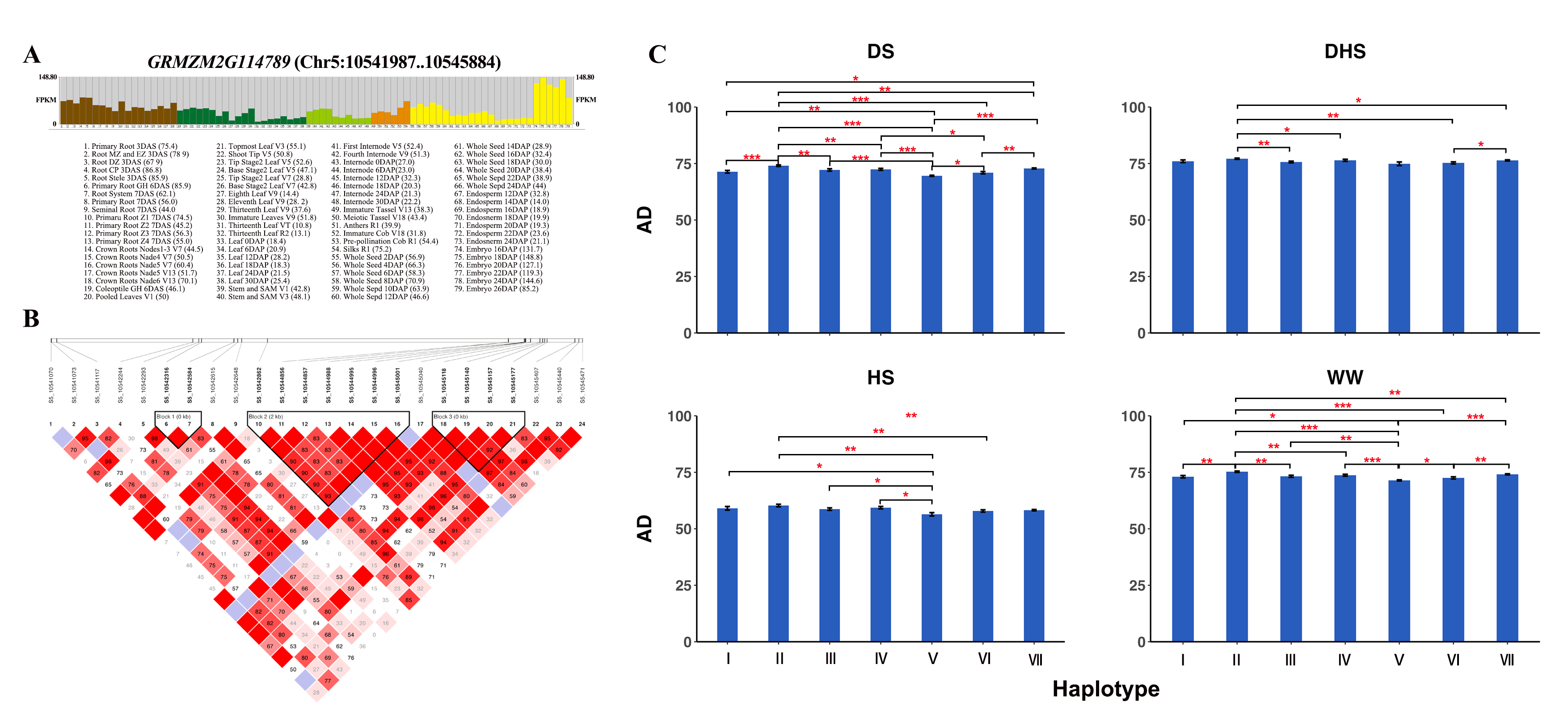

Supplement: Supplementary Figure 6 — (A) Tissue-specific expression profile, (B) Linkage disequilibrium, and haplotype block with 13 SNPs inside for the candidate gene GRMZM2G114789.. (C) Comparison of trait AD among haplotypes I (CCGGCCCAACACT), II (CCGGCCCAAGGCT), III (CCGGCCCAAGGTT), IV (TCGGCCCAACACT), V (TCGGCCCAAGGCT), VI (TCGGCCCAAGGTT), and VII (TCGGCTTCAGGTT). The number of stars represents the result of t test at different significance levels (*: 0.05; **: 0.01; ***: 0.001). [file Image_6.tif]
